# Activity and Coupling to Hippocampal Oscillations of Median Raphe GABAergic Cells in Awake Mice

**DOI:** 10.3389/fncir.2021.784034

**Published:** 2021-12-17

**Authors:** Marta Jelitai, Albert M. Barth, Ferenc Komlósi, Tamás F. Freund, Viktor Varga

**Affiliations:** ^1^Subcortical Modulation Research Group, Institute of Experimental Medicine, Budapest, Hungary; ^2^Laboratory of Cerebral Cortex Research, Institute of Experimental Medicine, Budapest, Hungary

**Keywords:** GABA, median raphe, *in vivo* awake patch-clamp, theta oscillation, hippocampal ripple

## Abstract

Ascending serotonergic/glutamatergic projection from the median raphe region (MRR) to the hippocampal formation regulates both encoding and consolidation of memory and the oscillations associated with them. The firing of various types of MRR neurons exhibits rhythmic modulation coupled to hippocampal oscillatory activity. A possible intermediary between rhythm-generating forebrain regions and entrained ascending modulation may be the GABAergic circuit in the MRR, known to be targeted by a diverse array of top-down inputs. However, the activity of inhibitory MRR neurons in an awake animal is still largely unexplored. In this study, we utilized whole cell patch-clamp, single cell, and multichannel extracellular recordings of GABAergic and non-GABAergic MRR neurons in awake, head-fixed mice. First, we have demonstrated that glutamatergic and serotonergic neurons receive both transient, phasic, and sustained tonic inhibition. Then, we observed substantial heterogeneity of GABAergic firing patterns but a marked modulation of activity by brain states and fine timescale coupling of spiking to theta and ripple oscillations. We also uncovered a correlation between the preferred theta phase and the direction of activity change during ripples, suggesting the segregation of inhibitory neurons into functional groups. Finally, we could detect complementary alteration of non-GABAergic neurons’ ripple-coupled activity. Our findings support the assumption that the local inhibitory circuit in the MRR may synchronize ascending serotonergic/glutamatergic modulation with hippocampal activity on a subsecond timescale.

## Introduction

“Timing is everything” is declared by one of the most famous quotes. Brain oscillations are expressive examples underscoring the validity of this claim. The synchronous activity of neurons is indispensable for communication within and across neural networks and for the plastic changes of their connections (Buzsáki and Draguhn, 2004; Paulsen and Sejnowski, 2006). Brain states coupled with disjunct stages of information processing, i.e., acquisition and consolidation are characterized by different oscillations. During exploration, when unfamiliar stimuli are encountered, the highly regular theta oscillation dominates brain circuits engaged in encoding the novel information (Vanderwolf, 1969). The same circuits switch to a distinct mode of operation, whereas the newly acquired information is being consolidated, marked by large amplitude waves cooccurring with high frequency transients termed sharp wave ripples (Buzsáki et al., 1992). The hippocampus and connected regions form the core of the information storing circuit and subcortical modulators are key regulators of its operational state. Serotonergic/glutamatergic pathways from the median raphe nucleus, one of the major nuclei of the ascending serotonergic system, have profound effects on hippocampal activity and hippocampus-dependent behaviors (Nitz and McNaughton, 1999; Varga et al., 2009; Wang et al., 2015; Szőnyi et al., 2019). The majority of raphe neurons’ activity fluctuates in concert with alternating brain states (Jacobs and Azmitia, 1992; Sakai and Crochet, 2001). However, in recent years, a considerable proportion of both serotonergic and non-serotonergic neurons were shown to exhibit rhythmic discharge synchronized to the hippocampal theta rhythm, and some of these cells’ firing probability changed during ripples (Prisco et al., 2002; Kocsis et al., 2006). A key, yet unanswered question, is how an oscillatory activity is imposed on modulatory neurons. In many regions, the orchestration of neuronal activity during the emergence of oscillations relies on rhythmic inhibition (Wang, 2010). In the midbrain raphe complex, besides forming a feedback circuit excited by locally released serotonin (Boothman and Sharp, 2005), GABAergic neurons collect forebrain inputs and convert them to a fluctuating inhibitory tone (Varga et al., 2003). Here, we aim to characterize the state-dependent activity and coupling to the major hippocampal oscillations of MRR GABAergic cells, whereby the inhibitory network of the median raphe may be capable of coupling ascending serotonergic/glutamatergic modulation to the hippocampal rhythmic patterns.

## Materials and Methods

### Animal Care and Housing

Male 8–12-week old, vGAT-iRES-Cre (Jackson Laboratory, JAX:016962) and vGAT-IRES-Cre/Gt(ROSA)26Sor_CAG/ZsGreen1 mice were used in this study. Gt(ROSA)26Sor_CAG/ZsGreen1 mouse strain originates from Jackson Laboratory, JAX:007906. Mice, two to three animals per cage, were housed on a reversed 12-h light/dark cycle. Food and water were available *ad libitum*. All experiments were approved by the Animal Care and Use Committee of the Institute of Experimental Medicine and the Committee for Scientific Ethics of Animal Research of the National Food Chain Safety Office under the project number PE/EA/200-2/2020 and were performed according to the 2010/63/EU Directive of the EC Council. All efforts were made to minimize pain and suffering and to reduce the number of animals used.

### Surgical Procedures

Virus injections were performed under general anesthesia by intraperitoneal injection of ketamine–xylazine mixture (dose by body weight, 100 and 10 mg/kg, respectively), and analgesic (Buprenorphine 0.1 μg/g, s.c.) was applied at the beginning of surgery. MRR region was targeted *via* a burr hole (AP, –4.1 mm, ML, 0.0 mm) perpendicular to the skull surface using a standard stereotaxic frame (David Kopf Instruments, Tujunga, CA, United States). AAV-EF1a-DIO-hChR2(H134R)-eYFP (UNC Vector Core, Chapel Hill, NC, United States) or AAV-CAG-FLEX-ArchT-GFP (UNC Vector Core, Chapel Hill, NC, United States) was injected at a depth of 4.6–4.5 mm from the skull surface through a pulled glass micropipette using Nanoject II precision microinjector pump (Drummond, Broomall, PA, United States). After injection, mice were allowed to recover for at least 2 weeks to ensure sufficient expression of the virus.

Surgeries for whole cell patch-clamp recordings were done under isoflurane anesthesia (0.5–1.5%), and analgesic (Buprenorphine 0.1 μg/g, s.c.) was applied at the beginning of surgery. A small lightweight headplate was attached to the skull using Optibond adhesive (Kerr, Brea, CA, United States) and Paladur dental acrylic (Kulzer, Hanau, Germany). During whole cell recordings, mice were head-restrained with a downward tilted head position (pitch angle: 20°). For optogenetic experiments, an optic fiber (100 μm core diameter, Thorlabs GmbH, Newton, NJ, United States) was implanted above MRR (AP, –5.4 mm, ML, 0.9 mm at 18° ML angle) and fixed with dental acrylic. On the day of recording, at least 2 days after headplate surgeries, craniotomies were drilled above MRR (AP, –5.6 mm, ML, –0.7 mm, patch electrode ML at 10° angle) and dorsal hippocampus (AP, –3.2 mm, ML, –2.2 mm, LFP electrode AP, 20° ML at 10° angle). The craniotomies were covered with fast sealant (Body Double, Smooth-On, Easton, PA, United States), and recording was performed at least 1 h after surgery to allow recovering from anesthesia.

Headplate surgeries for multichannel recordings were done under isoflurane anesthesia (0.5–1.5%), and analgesic (Buprenorphine 0.1 μg/g, s.c.) was applied at the beginning of surgery. A small lightweight headplate was attached to the skull using Optibond adhesive (Kerr, Brea, CA, United States) and Paladur dental acrylic (Kulzer, Hanau, Germany). During multichannel recordings, mice were head-restrained with a downward tilted head position (pitch angle: 20°). Two cranial windows (1.5 × 1.5 mm) were drilled above the left hippocampus (AP, –2.5 mm; ML, 2.5 mm) and MRR (AP, –6.1 mm; ML, 0.0 mm, MRR probe ML at 4° angle) under stereotaxic guidance. For the optic fiber and ground electrode a hole was drilled above MRR (AP, –6.1 mm; ML, 0.0 mm) and cerebellum (AP, –5.6 mm; ML, 2.0 mm) respectively. The craniotomies and drill holes were covered with fast sealant (Body Double, Smooth-On, Easton, PA, United States). After surgery, the mice were continuously monitored until recovered, and then they were returned to their home cages for at least 48 h before starting habituation to the head restraint.

### Whole Cell Patch-Clamp Recordings

Mice were habituated to the head restraint and experimental setup for 1–2 days before each recording session. Head restrained mice were free to run, walk, or sit on the treadmill equipped with a 2-m-long belt. Current-clamp recordings were performed from MRR, 4,200–4,800 μm from the pial surface using a Multiclamp 700B amplifier (Molecular Devices, CA, United States). The data were digitized using CED Micro 1401 laboratory interface (Cambridge Electronic Design Limited, Cambridge, United Kingdom) and recorded using Spike 2 acquisition software. No bias current was applied during recordings and series resistances ranged between 20 and 60 MΩ. Once we got a stabilized recording, optical stimulations (light source: 447 nm diode or 593 nm DPSS laser, Roithner Lasertechnik GmbH, Austria, Ikecool, Corporation, United States, respectively, the latter no longer operational) were applied following 1–2 min of the control period. Both whole cell patch-clamp and cell-attached recordings were stabile during quiet wakefulness but we abruptly lost the cells at the onset of movement. For the analysis, we selected periods with stabile membrane potential; thus, the usual recording length was 150–250 s (median 181 s, interquartile range 99.5–301 s) with the shortest period of 30 s (Supplementary Table 1). For anesthetized experiments urethane (0.007 mL/g of 20%) was ip. administered at the beginning of the recording. *In vivo* external solution contained (in mM) 150 NaCl, 2.5 KCl, 10 HEPES, 1.5 CaCl_2_, and 1 MgCl_2_ (pH 7.3). Patch pipettes (5–8 MΩ) were filled with (in mM) 125 K-gluconate, 7 KCl, 10 HEPES, 10 sodium phosphocreatine, 0.1 EGTA, 2 MgATP, 2 Na_2_ATP, 0.5 Na_2_GTP (pH 7.2, 280–295 mOsm), and 2 mg/mL biocytin was added before recording. At the end of the recording, mice were transcardially perfused with 4% paraformaldehyde (PFA) and the brain was removed for *post-hoc* immunohistochemistry.

### Multichannel Electrophysiological Recordings

Mice were habituated to head restraint and experimental setup for 1–2 days. Head-restrained mice were free to run, walk, or sit on air supported free-floating 20 cm diameter polystyrene ball. On the day of recording Buzsaki64 or Poly5 silicon probes (Neuronexus, Ann-Arbor, MI, United States) were lowered through the cranial window to the left dorsal hippocampus and MRR under isoflurane anesthesia (0.75–1.5%). The optical fiber was inserted in the MRR at 4.1 mm depth from the skull surface. Probes and optical fiber were coated with a lipophilic fluorescent dye, DiI (Thermo Fisher Scientific, Waltham, MA, United States), for later histological verification of the location. A ground electrode was placed above the cerebellum. Mice were allowed to recover from anesthesia for ∼1 h before recording.

The probe in the hippocampus was advanced using a micromanipulator (David Kopf Instruments, Tujunga, CA, United States) until the pyramidal layer was detected by increased unit activity and the occurrence of ripple events. In the MRR, unit activity was monitored from 4.2 to 5.2 mm depth, and the final position was determined based on the appearance of units ceasing their firing upon 2 s long laser stimulation (593 nm, Ikecool Corporation, United States). Once we found optically silenced units, the recording was commenced after an approximately 1 h waiting period for letting the tissue settle around the probe. Electrophysiological recordings were performed by a signal multiplexing head-stage (RHD 128, Intan Technologies, LA, United States) and an OpenEphys data acquisition board^1^. Signals were acquired at 20 k sample/s (Open Ephys 0.4.4.1). Mouse locomotor activity was monitored with an optical computer mouse positioned close to the polystyrene ball at the equator. Speed was calculated by a custom written macro in Igor Pro. At the end of the recording, mice were transcardially perfused with 4% PFA and the brain was removed for *post-hoc* immunohistochemistry.

Neuronal spikes were detected and automatically sorted by a template-matching algorithm using the Spyking Circus software (Yger et al., 2018), followed by manual curation of the clusters using the Phy software (Rossant et al., 2016) to obtain well-isolated single units. Spike sorting quality was assessed with a refractory period violation, and visual inspection of auto- and crosscorrelations; poor quality clusters were discarded.

### Cell Identification

The location of the recorded cell was confirmed by biocytin labeling. We analyzed only those recordings in which the recording electrode, visible by the non-specifically labeled biocytin track, was inside the MRR region. Out of 108 recorded cells, we obtained 27 whole cell recordings. Of these, 13 were unequivocally identified as GABAergic or non-GABAergic. If we had been not able to find the biocytin labeled cell or there were more, equally labeled cells, we classified the registered neurons as non-identified (NI). The neurotransmitter phenotype of the recovered biocytin-labeled cells was determined by the colocalization of ZsGreen1 (selectively expressed by GABAergic neurons in the ZsGreen reporter mice, see above) or alternatively, by *post hoc* immunohistochemistry.

Optotagging was also used for cell identification. In AAV-EF1a-DIO-hChR2(H134R)-eYFP-injected vGAT-ires-Cre mice, if light stimulation (447 nm) had evoked depolarization and spiking, or hyperpolarization and spike suppression, the cell was identified as GABAergic or non-GABAergic, respectively. If the delivery of 593 nm light in AAV-CAG-FLEX-ArchT-GFP-injected vGAT-ires-Cre mice silenced the cell or evoked marked depolarization, the neuron was classified as GABAergic or non-GABAergic, respectively.

### Immunohistochemistry

For the reconstruction of neuronal morphology and probe locations, coronal or parasagittal sections (60 μm) were cut using a vibratome (Leica Microsystems, Wetzlar, Germany). Sections were first incubated in blocking solution for 2 h (10% normal goat serum (NGS), 0.5% Triton X-100 in 0.1 M phosphate-buffered saline (PBS), and then incubated overnight in streptavidin AlexaFluor-488 or Cy3 (1:1,000, Jackson Immuno Research) to reveal the identity of the biocytin-filled neuron. To amplify the signal of the virally delivered fluorescent reporter protein, coexpressed with ChR2 or ArchT, sections from transduced brains were incubated in chicken anti-Green Fluorescent Protein (GFP, 1:2,000, Life Technologies) primary and anti-chicken AlexaFluor-488 secondary antibodies (1.1,000, Thermo Fisher Scientific). 5-HT and vGlut3 content were investigated by incubating the sections in guinea pig anti-vGlut3 (1:1,000, Frontier Institute, Nittobo Medical Co.) and rabbit anti-5HT (Immunostar) primary and anti-guinea pig AlexaFluor-647 or AlexaFluor-405 and anti-rabbit AlexaFluor-647 or 405 (1:1,000, Thermo Fisher Scientific or Jackson Immuno Research). All antibodies were diluted in carrier solution (0.1 M PBS, 1% NGS, 0.2% Triton-X100) and incubated overnight at room temperature. Fluorescence signals (DiI, streptavidin Alexa, or fluorescent reporter protein of ChR2, ArchT, and vGAT) were inspected using an Axioplan 2 microscope (Carl Zeiss, Oberkochen, Germany). To investigate the neurochemical content (vGlut3, 5-HT, vGAT) of biocytin-labeled cells, *z* stack images were acquired using a Nikon C2 confocal microscope (x40 objective, Nikon, Europe).

### Data Analysis

All *in vivo* data were analyzed in Igor Pro 8 (Wavemetrics, Lake Oswego, OR, United States). Action potentials from whole cell recording voltage traces were detected automatically using an amplitude threshold algorithm (TaroTools, custom written macro in Igor Pro)^2^ and were manually verified. The membrane potential spike threshold was computed as the second derivative of the voltage trace. Average membrane potential was calculated after clipping spikes above the threshold voltage. To calculate theta phase coupling, recording channels with maximal ripple amplitude, corresponding to the pyramidal layer, were selected. LFPs were filtered in the theta range (4–12 Hz). The phase of the theta oscillation was calculated by computing the Hilbert transform. Each single unit spike had been assigned to its coincident phase value and then the phase distribution was computed. Theta-coupling was assessed by performing Rayleigh’s test for circular uniformity (*p* > 0.05). For computing theta rhythmicity index, autocorrelograms of every single unit were calculated, and theta rhythmicity index was defined as the ratio of the local minimum and maximum in a time window between 80 and 200 ms. To investigate the correlation between firing frequency and speed, the latter was normalized by its maximum in every animal and then divided into 25 bins. The firing rate of the neuron in each speed bin was then plotted and the resulting point cloud was fitted by linear regression. To analyze ripple coupling, hippocampal recording channels with maximal ripple amplitude were selected and then downsampled to 1 kHz. The LFPs were band-pass filtered in the 120–250 Hz range and the Hilbert transform was computed. Ripples were detected as events exceeding 3 SD of the signal with a minimal duration of 20 ms. The *z*-scored ripple peak triggered perievent histograms (PETH) were created for every single unit. Fast ripple-coupled units were defined if the *z*-scored PETH exceeded 2 SD in either the positive or negative direction for at least 20 ms in a 300 ms window around ripple peak. Slow reduction of ripple-coupled firing suppression was defined as a > 2 SD decrease of the *z*-scored PETH values for at least 500 ms in a 5-s-long window preceding the ripple peak. The onset and offset of ripple-coupled activity were defined as the 1 SD crossings. For the stimulus-triggered wavelet, the LFP was subjected to continuous wavelet transformation, utilizing a Morlet mother wavelet.

In whole cell experiments in each session shuffled PETHs were generated by selecting random timepoints matching the number of ripples in that session repeated 100 times. The ripple-triggered PETHs were plotted with the corresponding 95% confidence bounds of the shuffled data. If bin values exceeded the 95% confidence bounds for at least 20 ms, it was defined as a significant deviation.

All statistical analyses were performed with standard Igor Pro 8 functions. For comparisons, Student’s *t*-test or Mann–Whitney and Wilcoxon test were used. The value *p* < 0.05 was considered significant. All values indicate mean ± SD unless stated otherwise.

## Results

### Heterogeneous GABAergic Population in Median Raphe Region

**FIGURE 1:**
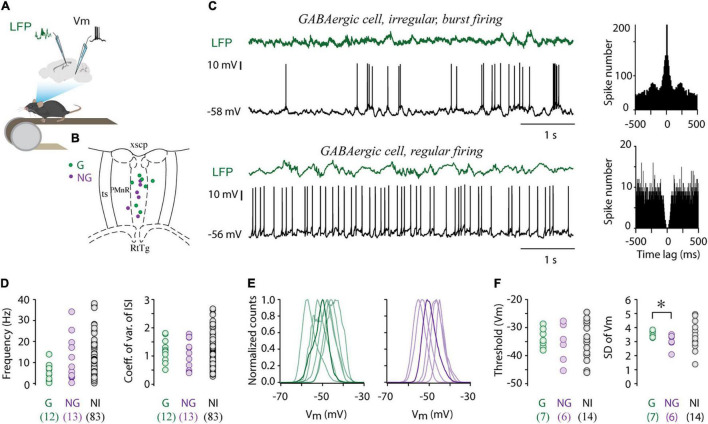
Electrophysiological properties of MRR GABAergic cells. **(A)** Schematic showing the recording configuration. Whole cell recordings were collected from the MRR, simultaneously with local field potentials (LFP) from the dorsal hippocampal CA1 region in head-fixed behaving mice. **(B)** Colored dots (G: GABAergic, NG: non-GABAergic) mark the position of whole cell recorded and neurochemically identified neurons overlaid onto representative coronal diagrams of MRR (median raphe and paramedian raphe region). RtTg, reticulotegmental nucleus of the pons; PMnR, paramedian raphe nucleus; ts, tectospinal tract; xscp, decussation of the superior cerebellar peduncle. **(C)** Representative examples of whole cell current-clamp recordings and associated LFP (green) of an irregular (top trace) and regular (lower trace) GABAergic cell. Associated autocorrelograms are on the right sides. **(D)** Average firing rates and coefficient of variations of inter-spike intervals of GABAergic cells (G, *n* = 12), identified non-GABAergic (NG, *n* = 13) cells and non-identified cells (NI, *n* = 83). Open circles represent individual MRR neurons. **(E)** Normalized membrane potential (Vm) distribution of seven GABAergic (left) and six non-GABAergic (right) cells, thick lines highlight a single typical Vm distribution. **(F)** Average action potential threshold and standard deviation of membrane potential (SD of Vm) of GABAergic cells (G, *n* = 7), identified non-GABAergic (NG, *n* = 6) cells and non-identified cells (NI, *n* = 14). Circles represent individual MRR neurons. *p < 0.05, two-tailed *t-*test.

To uncover the physiological characteristics and state-dependent activity of GABAergic and non-GABAergic neurons in the median raphe nucleus and surrounding paramedian raphe [together termed median raphe region (MRR)], we performed whole cell patch-clamp or cell-attached recordings from MRR with the simultaneous registration of local field potentials (LFP) in the hippocampal CA1 region in head-fixed behaving mice (Figure 1A). LFP was recorded from the pyramidal layer of dorsal CA1, identified based on the occurrence of ripple waves during immobility. The location of the recorded cell was confirmed by biocytin labeling. We analyzed only those sessions in which the recording electrode (visible by the biocytin-labeled track) was inside the MRR (Figure 1B).

We obtained 27 whole cell recordings and 81 cell attached recordings (*N* = 62 mice) from the MRR in single cell recording experiments. There was no difference in the firing frequency (median 4.72 Hz, interquartile range 2.59–8.71 Hz vs. median 7.45 Hz, interquartile range 3.21–14.09 Hz, Wilcoxon signed-rank test) nor in the coefficient of variation of inter-spike intervals (CV_*ISI*_, median 1.06, interquartile range 0.79–1.35 vs. median 1.13, interquartile range 0.80–1.66, Wilcoxon signed-rank test) between whole cell and cell attached recordings.

For the identification of GABAergic cells, we have used a double transgenic mouse line, generated by crossing ZsGreen1 fluorescent reporter mice with vesicular GABA transporter (vGAT)-Cre mice, in which GABAergic cells expressed the ZsGreen fluorescent protein. Non-GABAergic neurons were labeled *post hoc* for vesicular glutamate transporter 3 (vGlut3) and serotonin (5HT). The unequivocal neurochemical classification was possible in 25 out of 108 recorded MRR cells: 12 cells were GABAergic whereas 13 cells were proved to be non-GABAergic (NG). In the remaining 83 cells, the neurotransmitter phenotype could not be determined (non-identified cells, NI). Firing frequency varied widely in each group, from 0.33 to 13.9 Hz in GABAergic cells (*n* = 12, median 5.78 Hz), 0.35–34.18 Hz in non-GABAergic neurons (*n* = 13, median 5.54 Hz), and between 0.18 and 38.16 Hz in case of NI neurons (*n* = 83, median 7.41 Hz, Figure 1D but see Supplementary Table 1). Accordingly, both GABAergic and non-GABAergic cells displayed a broad range of ISIs without significant difference in their distribution (median 145.95 ms, interquartile range 118.12–457.87 ms vs. median 179.70 ms, interquartile range 58.33–315.30 ms, Wilcoxon signed-rank test). The firing pattern regularity of GABAergic neurons formed a continuum from regular to irregular reflected in the broad distribution of CV_*ISI*_ (median 1.18, range 0.51–1.81. Figures 1C,D). The majority of these cells typically displayed irregular firing patterns with intermittent bursting (Figure 1C), sharply distinct from the characteristic clock-like, tonic, serotonergic firing pattern (Aghajanian et al., 1978; Prisco et al., 2002; Kocsis et al., 2006). The resting membrane potential of GABAergic cells varied between –44.02 and –55.46 mV (*n* = 7, –49.50 ± 4.03 mV) and they displayed unimodal subthreshold Vm distribution, centered around –50 mV (Figure 1E). The membrane potential distribution of GABAergic and non-GABAergic cells (*n* = 6, –48.70 ± 4.03 mV, two-tailed *t-*test) was not statistically different. Action potential threshold of GABAergic cells (–33.77 ± 3.42) was also in the same range as that of non-GABAergic neurons (–35.6 ± 6.88, Figure 1F). However, large subthreshold Vm fluctuations characterized the former population thus, the standard deviation (SD) of Vm of GABAergic and non-GABAergic cells was significantly different (3.56 ± 0.22 vs. 3.04 ± 0.52, two-tailed *t-*test, *p < 0.05, Figure 1F).

### Tonic Inhibition of Non-GABAergic Projection Neurons by Local GABAergic Cells

To directly investigate the effect of local GABAergic cells on the spike output of non-GABAergic putative projection neurons (Jacobs and Azmitia, 1992; Hioki et al., 2009; Jackson et al., 2009; Varga et al., 2009; Szőnyi et al., 2014, 2019), we combined cell type-selective optogenetic manipulation of the former with whole-cell patch clamp recordings from MRR neurons. In several cases, we abruptly lost the cells at the middle of the stimulation because of movement, therefore some of the experiments (*n* = 6/6 at ChR2 and *n* = 3/11 at ArchT) were carried out under urethane anesthesia. Channelrhodopsin 2 (ChR2) was targeted to MRR cells using the vGAT-Cre transgenic mouse line and viral gene transfer (AAV vector). One millisecond-long optogenetic single pulse stimulation did not evoke a detectable effect in the membrane potential but lengthening the pulse to 3 or 10 ms resulted in marked depolarization or spiking in GABAergic cells (*n* = 3) and phasic hyperpolarization (–3.36 and –5.39 mV, respectively) in non-GABAergic cells (*n* = 3) (Supplementary Figure 1).

**FIGURE 2:**
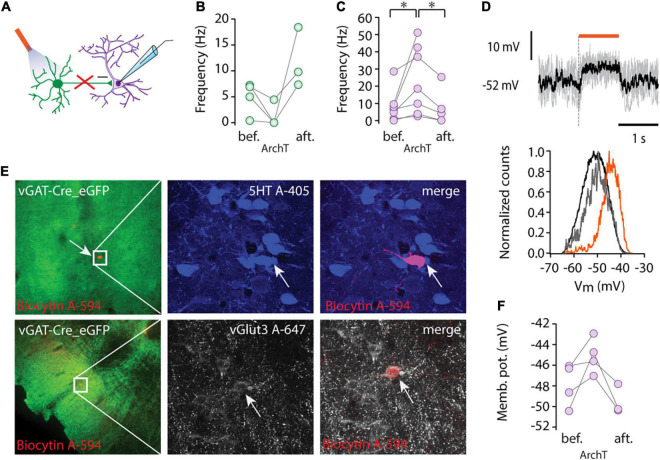
Tonic inhibition by local GABAergic neurons. **(A)** Schematic showing recording configuration during optogenetic stimulation of GABAergic cells (ArchT, 593 nm). **(B)** Average firing rate of GABAergic cells (*n* = 4) before (bef.), during light stimulation (ArchT), and after light stimulation (aft.). **(C)** Average firing rate of non-GABAergic cells (*n* = 7) before (bef.), during light stimulation (ArchT), and after light stimulation (aft.), *p < 0.05, Wilcoxon signed-rank test. **(D)** Membrane potential of a representative non-GABAergic cell (upper panel) during light stimulation (orange bar, 1s pulse of 593 nm light stimulation), five consecutive traces are overlaid (gray) and the black trace shows the average Vm. Representative normalized Vm distribution (lower panel) before light stimulation (black), during light stimulation (orange) and after light stimulation (gray). **(E)** Lower (left column) and higher magnification immunofluorescent images (middle, right columns) of representative recorded cells labelled by biocytin (red) from virus injected vGAT-Cre mice (ArchT-eGFP, green). One cell was serotonin positive (5HT, blue, top row) and the other one was immunoreactive for vGlut3 (white, lower row). **(F)** Average membrane potential of four non-GABAergic cells before (bef.), during light stimulation (ArchT) and after light stimulation (aft.).

ArchaerhodopsinT [ArchT, Han et al. (2011)] was utilized for suppressing neuronal activity (Figure 2A). To assess the efficiency of ArchT, we recorded from GABAergic neurons and found that illumination (5 s pulse, 593 nm) caused marked hyperpolarization (delta Vm: –10.9 ± 12.32 mV, *n* = 3) and silencing of three out of four tested cells (*n* = 4, Figure 2B).

Inactivation of the GABAergic circuit in the MRR revealed a marked tonic inhibition of non-GABAergic projection neurons. ArchT mediated suppression (1–2 s pulses) of GABA-mediated inhibition caused depolarization and a rightward shift in Vm distributions in the neighboring, non-GABAergic cells (Figure 2D). The membrane potential depolarization (from –47.87 ± 2.07 mV to –45.08 ± 1.72 mV, *n* = 4, Figure 2F) was only observed during light stimulation and returned to baseline after stimulus cessation (onset and offset latencies were 30.72 ± 3.17 ms and 38.64 ± 14.98 ms, respectively). Depolarization during the suppression of local inhibition was paralleled by a marked increase in non-GABAergic cells’ firing rates (*n* = 7, from 0.88 to 3.66 Hz, median, *p < 0.05, Wilcoxon signed-rank test, Figure 2C). After the termination of light delivery, a small rebound hyperpolarization (–3.36 ± 2.39 mV) was observed in the majority of recorded non-GABAergic units (*n* = 3/4).

Among the intracellularly recorded neurons (*n* = 4), we found one 5HT positive/vGlut3 negative cell, one vGlut3 positive neuron (Figure 2E), and a putative vGlut2 positive cell, which was negative for both 5HT and vGlut3. These results confirm that each major type of non-GABAergic projection neuron in the MRR is under a tonic inhibitory control by local GABAergic cells.

### State Dependent Firing of Median Raphe Region GABAergic Cells

**FIGURE 3:**
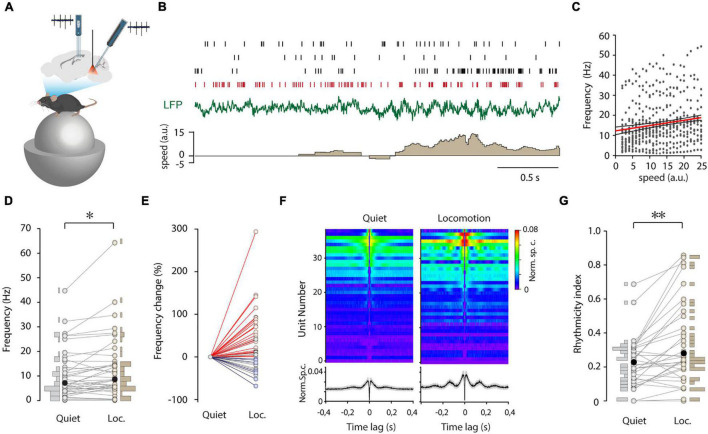
State-dependent firing of MRR GABAergic cells. **(A)** Schematic showing the recording configuration. Dual silicon probe recordings were collected from the median raphe region (MRR) and CA1 of the dorsal hippocampus in head-fixed, behaving mice. The optic fiber was placed above the MRR. **(B)** Representative traces with four concurrently registered GABAergic cells from one session. Spike raster plots (top), LFP (middle, green), and speed (lower, brown) during a non-theta to a theta state transition. Note the emergence of theta oscillation with movement (a.u., arbitrary units). Spikes of a typical theta rhythmic GABAergic cell are colored red. **(C)** Frequency of positively correlated GABAergic cells (21/39) as a function of normalized speed. The red line marks the linear fit with a 95% confidence interval (black lines). **(D)** Average firing rate of GABAergic cells (*n* = 39) and its distribution during quiet wakefulness (Quiet) and locomotion (Loc.) Black filled circles represent the median. *p < 0.05, Wilcoxon signed-rank test. **(E)** Change in firing rate during quiet wakefulness (Quiet) and locomotion (Loc.) normalized to a quiet state. Colored circles represent GABAergic cells that displayed an increase (red lines and markers), decrease (blue lines and markers), or no significant (gray) change in firing rate. For testing significance Wilcoxon-Mann-Whitney test, *p* < 0.05 was used. **(F)** Autocorrelograms of GABAergic units (*n* = 39) during quiet wakefulness (left) and locomotion (right). Autocorrelograms were ordered by average values between 3 and 20 ms during locomotion. Lower panels display the average autocorrelograms (black line and gray shadow: mean ± s.e.). **(G)** Paired plot of rhythmicity of GABAergic cells (*n* = 39) and its distribution during quiet wakefulness (Quiet) and locomotion (Loc.) Black symbols represent median. **p < 0.01, Wilcoxon signed-rank test.

State-dependence is a hallmark of neuronal activity in the median raphe, possibly shaped by the MRR GABAergic circuit. Hence, we compared the firing of MRR GABAergic cells during non-theta and theta states. Due to microwobbling amplified by the proximity of the basilar artery and the central aquaeduct to the MRR, whole cell recording was possible only during immobility (*n* = 27 of 108 neurons). Nevertheless, short theta segments could be isolated in 15 cells (*n* = 15/27), and the membrane potential of GABAergic cells was found to be unchanged while transient theta epochs emerged compared to the non-theta state (Supplementary Figure 2A). To study state-dependence in a larger population of GABAergic neurons, we deployed multichannel recording combined with inhibitory optical tagging (Figure 3A). ArchT was targeted to MRR GABAergic cells by viral gene transfer in vGAT-Cre mice. MRR and hippocampal activity were simultaneously sampled by silicone probes in head-fixed, awake animals running or sitting on air-supported free-floating (20 cm diameter) polystyrene balls. Delivery of 2-s long laser pulses enabled the tagging of even low activity cells (Supplementary Figure 2B). We could identify 39 GABAergic units from five animals. Their firing frequency varied in a wide range from 0.06 to 24.29 Hz (median 5.08 Hz) and did not differ significantly from the frequency of GABAergic neurons recorded by the whole cell patch-clamp (Supplementary Figure 2C). The appearance of theta was accompanied by the change of firing activity in the majority of GABAergic neurons (Figure 3B). During locomotion, the firing frequency increased, decreased, or remained unaffected in *n* = 23/39, 7/39, and 9/39 cells, respectively (Figures 3D,E). The direction of firing rate modulation did not depend on the magnitude of firing rate during quiet wakefulness (*r* = –0.114, *p* = 0.487), but GABAergic cells varied in their sensitivity to movement. That is, 21/39 GABAergic cells displayed a firing rate increase that was positively correlated with speed (Figure 3C), 16/39 cells showed no correlation, whereas 2/39 cells were inversely correlated. Besides the level of activity, we also explored how the firing pattern regularity of these neurons is affected by state transitions. Various patterns ranging from regular to irregular were observed spreading along a continuum reflected in the broad distribution of CV_*ISI*_ (Supplementary Figure 2C). Non-theta to theta state transition at the onset of movement altered the firing pattern, resulting in augmented rhythmicity and the occasional clustering of spiking (Figures 3B,F). In addition, there was no correlation between the rhythmicity during quiet wakefulness and the magnitude of the rhythmicity change (*r* = –0.223, *p* = 0.237), albeit the more rhythmic the cells were, the larger increase of frequency was detected during locomotion (*r* = 0.505, *p* = 0.004). Interestingly, there was an abrupt increase of theta power in the hippocampus with accompanying suppression of subtheta bands parallel with the light stimulation when the majority of MRR GABAergic cells were presumably silenced (Supplementary Figure 3). Importantly, the elevation of theta frequency preceded the onset of movement.

### Hippocampal Theta and Sharp Wave Ripple-Coupled Firing of Median Raphe Region GABAergic Neurons

**FIGURE 4:**
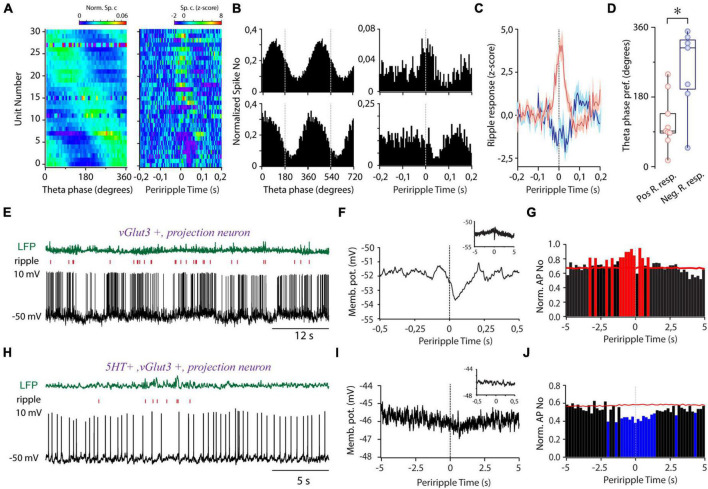
Theta and sharp wave ripple-coupled firing in the MRR. **(A)** Preferred theta phase ordered phase histograms (left panel) (*n* = 31) and the corresponding periripple firing histograms of theta coupled GABAergic cells. **(B)** Two representative examples demonstrating theta- (left panels) and ripple- (right panels) coupled firing of GABAergic cells (bin size 5 ms). **(C)** Averaged periripple firing histograms of GABAergic cells with fast transient elevation (red, *n* = 8) and fast transient suppression (blue, *n* = 7) during ripples. **(D)** Theta phase preference of cells with positive transient (Pos. R resp., pink, *n* = 8) and negative transient (Neg. R. resp., blue, *n* = 7) during ripple waves. Circles represent individual GABAergic cells, boxes denote the medians and interquartile ranges. For testing significance, Wheeler-Watson and Whatson-Williams tests, *p < 0.05 were used. **(E)** LFP (green, top), ripple raster plot (red, middle), and voltage trace (lower panel) from a non-GABAergic ripple-coupled cell. **(F)** The membrane potential of the cell **(E)** as a function of periripple time. Inset: Ripple peak aligned membrane potential at second-long timescale. **(G)** Normalized periripple firing histogram (bin size = 200 ms) of the cell **(E)**. The red color denotes the significant increase in the firing rate. **(H)** LFP (green, top), raster plot (red, middle), and voltage trace (lower panel) from a non-GABAergic ripple suppressed cell. **(I)** The membrane potential of the cell **(H)** as a function of periripple time on a slow timescale. Inset: ripple peak aligned membrane potential on a subsecond timescale. **(J)** Ripple peak aligned averaged normalized firing histogram (bin size = 200 ms) of the cell **(H)**. The blue color denotes a significant decrease in the firing rate.

Next, we aimed to unravel the temporal relationship of the activity of MRR GABAergic cells to hippocampal theta. The majority of GABAergic cells showed theta phase-coupled firing (*n* = 31/39, Rayleigh test for uniformity, *p* < 0.05, mean resultant length 0.27 ± 0.02) covering the whole theta cycle as a population (Figure 4A). A subgroup of theta preferring MRR GABAergic neurons (*n* = 16/31, Figures 4A,B) exhibited ripple correlated-activity (absolute *z*-score values higher than 2 for at least 20 ms in a 300-ms window around ripple peak). Ripple activated cells covered mostly the ascending phase of theta, whereas cells with fast transient suppression of activity during ripples concentrated near the trough (Figure 4D). Transient activation (*n* = 9/31) lasted about 52 ms on average (51.67 ± 17.5 ms) centered around ripple peaks. On average, rapid suppressions (*n* = 7/31, 46.43 ± 22.3 ms) tended to start later compared with activation relative to ripple peaks (23.89 ± 11.11 vs. 10.71 ± 24.57 ms before ripple peaks (Figure 4C), but the difference did not reach significance. Ripple-associated transient increase of activity was also observed in two cells (*n* = 2/8) with no theta preference (*n* = 8/39) (Supplementary Figure 4A). Ripple-coupled fast transient membrane potential changes could not be observed in the identified whole cell recorded GABAergic cells (*n* = 0/7).

### The Ripple-Correlated Output of Median Raphe Region

We aimed to explore if alteration of local inhibition during ripples is mirrored by a complementary change in the ripple-coupled firing of the MRR’s non-GABAergic output neurons. To this end, we investigated the ripple-correlated firing of these neurons recorded in whole cell mode.

One glutamatergic cell (5HT-, vGlut3+) showed fast transient suppression of spiking (Figure 4E) after ripple peak paralleled by a marked membrane potential hyperpolarization, reaching its maximum postpeak (Figure 4F). In the background of the rapid hyperpolarizing transient, we could also detect a slow depolarization on multiple second-long timescales, resulting in a significant elevation of activity around ripples (Figure 4G). In a serotonergic/glutamatergic cell (5HT+, vGlut3+), we observed slow gradual hyperpolarization and simultaneous decrease of activity concurrently with ripples (Figures 4H–J), but without any detectable ripple-locked fast transient. The diverging timescales and opposite direction of activity change suggest that different mechanisms govern the ripple-associated activity of MRR neurons.

The observed second-long ripple-correlated activity change prompted us to investigate the ripple-associated activity at a longer timescale in the identified GABAergic group. One whole cell recorded GABAergic cell showed slow hyperpolarization, and 9 out of 39 GABAergic neurons suppressed their activity for 1.50 ± 1.19 s around ripples (Supplementary Figure 4B). Strikingly, the majority of these neurons (*n* = 6/9) exhibited fast transient ripple-coincident activation embedded in longer-lasting suppression thus, exhibiting contrasting activity change simultaneously on different timescales. We did not detect multiple second-long elevations of activity among GABAergic cells around ripples.

## Discussion

GABAergic neurons form the largest subgroup in the median raphe nucleus and surrounding paramedian raphe (MRR). However, we know surprisingly little about this component of the raphe circuit. Here, by employing extracellular single or multichannel recording and whole cell patch-clamp in awake mice, we uncovered significant firing pattern heterogeneity, state-dependent activity, and tight coupling to hippocampal oscillations of MRR GABAergic cells. Utilizing whole cell patch clamp in awake animals let us observe the characteristics of inhibition in the most intact preparation.

Early *in vivo* pharmacological studies demonstrated the tonic control of serotonergic neurons in the MRR by GABAergic transmission (Forchetti and Meek, 1981). Later, slice experiments corroborated and extended these findings by characterizing GABAA-receptor mediated synaptic currents (IPSC) in serotonergic neurons of the midbrain raphe nuclei (Lemos et al., 2006). Comparison of IPSCs in serotonergic neurons of the MRR vs. the DRN exposed higher inhibitory activity in the former. These findings were further strengthened by the immunocytochemical and pharmacological detection of alpha3 subunit-containing GABAA receptors in serotonergic cells (Rodríguez-Pallares et al., 2001; Judge et al., 2006). A surprising report showed a higher than 90% colocalization of serotonin and GABAB-receptors in both the dorsal and median raphe nuclei, thus hinting about the tight regulation of the serotonergic output by both fast and long-acting GABAergic inhibition (Varga et al., 2002). In accordance with these preceding studies, we observed a powerful phasic inhibition of serotonergic and vesicular glutamate transporter type 3-expressing glutamatergic neurons in response to brief stimulation of GABAergic cells. Additionally, suppression of the inhibitory circuit uncovered the tonic inhibition of non-GABAergic units.

A series of *in vivo* pathway stimulation studies and recently, retrograde viral tracing in the dorsal raphe demonstrated that the GABAergic circuit is targeted by a large variety of raphe afferents (Wang and Aghajanian, 1977; Varga et al., 2001, 2003; Weissbourd et al., 2014) and may thus be in a key position to convert inputs to a complex, yet uncharacterized inhibitory pattern. For example, stimulation of the medial prefrontal cortex and the lateral habenula, two major raphe-projecting forebrain areas, evokes GABAA-mediated suppression of activity in the majority of putative serotonergic neurons (Wang and Aghajanian, 1977; Varga et al., 2001, 2003; Weissbourd et al., 2014). Besides distant projections, GABAergic neurons are influenced by locally released serotonin *via* 5-HT2A/C receptors, whereby they are key constituents of a local feedback circuit (Boothman and Sharp, 2005). The diversity of inputs is reflected by the irregularity of their firing as reported in a preceding study that utilized juxtacellular recording and labeling of dorsal raphe neurons in anesthetized rats (Allers and Sharp, 2003). We could show that irregular firing pattern was underlined by complex membrane potential dynamics reflected in its high variability in the undrugged animal.

Vigilance states are principal correlates of neuronal activity in many subcortical regions, including the median raphe (Lee and Dan, 2012). Furthermore, serotonergic cells, in concert with other subcortical modulators, were proposed to be major regulators of the sleep-wake cycle (Jacobs and Azmitia, 1992). Thus, sleep-wake stage-coupled discharge of serotonergic neurons is at least partly controlled by their interplay with other modulators. Here, we found that the activity of most MRR GABAergic neurons reliably followed behavioral activation, i.e., transition from immobile state to running. Since serotonergic neurons excite the local GABAergic circuit *via* 5-HT2A/C receptors, elevated serotonergic activity can turn up GABAergic inhibition as well. In turn, the latter can serve as a break synergistically with the autoinhibition of serotonergic neurons. Besides fluctuation on long time scales, we observed the coupling of GABAergic spiking to both of the disjunct hippocampal oscillatory patterns: theta rhythm and sharp wave ripples. An unexpected discovery revealed the serotonergic identity of previously described theta-coupled rhythmic raphe neurons (Kocsis et al., 2006). This finding went against the widely accepted view of regular, metronome-like firing pattern, as a defining characteristic of serotonergic cells (Aghajanian et al., 1978). Phase-locked activity, albeit weaker, was also observed in glutamatergic cells of the MRR (Domonkos et al., 2016). A key question concerned the source of this rhythm. Theta-modulated GABAergic neurons described in our study may be prime candidates for conveying theta drive from forebrain rhythm-generating networks, most notably the medial septum, to the serotonergic and glutamatergic neurons of the MRR. Timing neuromodulation with theta timescale precision would dramatically increase its computational capacity, thus, activity in memory and executive circuits can be tailored to moment-to-moment changes of a challenging behavioral situation. The range of preferred theta phases covering the entire cycle would raise the intriguing possibility of functional subgroups of GABAergic cells dividing neuromodulation in the temporal domain. This scenario would be analogous to the hippocampus where activity in principal cell compartments is temporally segmented by the various interneuron subtypes linked to different phase ranges of theta (Klausberger and Somogyi, 2008). Besides theta, MRR GABAergic cells also exhibited ripple-correlated activity. The role of hippocampal ripples in memory consolidation is supported by many elegant studies (Girardeau et al., 2009; Nakashiba et al., 2009; Fernández-Ruiz et al., 2019). The antagonism of cholinergic and serotonergic modulation and ripple genesis is also well-established (Vandecasteele et al., 2014; Wang et al., 2015; Zhang et al., 2021). As in the case of theta-coupling of serotonergic and glutamatergic MRR neurons, the question arises about mechanisms that suppress the activity of these modulatory cells during ripple genesis. In a recent study, selective activation of GABAergic MRR neurons failed to change the rate of ripple occurrence, but following the stimulation transient suppression of ripples ensued possibly due to the rebound activation of GABA-targeted serotonergic/glutamatergic neurons (Wang et al., 2015). In our experiments, about a two-third majority of GABAergic MRR cells were activated during ripples. The increase of their activity started before the ripple peak and returned to baseline around the end of ripples. This tight coupling to the ripple window is orders of magnitude faster than the slow disfacilitation of MRR cell activity reported in the aforementioned study and suggests that the rapid rise of inhibition may switch off its target modulators and opens a narrow window of opportunity for communication between the hippocampus and its connected cortical partners (e.g., Sanda et al., 2020). This assumption was supported by the rapid hyperpolarization during ripples of an intracellularly recorded glutamatergic neuron. As theta coupling, this mode of operation is radically different from the slow modulation, traditionally associated with the serotonergic and other subcortical modulators. Notably, the firing of the remaining one-third of GABAergic MRR neurons was dampened, but on a comparably short timescale as activation. Here, the yet unanswered question can be posed whether this subgroup of ripple-suppressed inhibitory cells may influence a different target population of non-GABAergic neurons than the facilitated cells. The complementary ripple coincident change of firing of activated and inhibited GABAergic neurons also points to mutual inhibition among these cells. Strikingly, activity during ripples was correlated with theta phase preference. This observation strengthens the possibility that multiple functional groups exist in the MRR’s inhibitory circuit capable of temporally segmenting neuromodulation depending not just on brain states, but also on momentary changes of activity patterns in target regions. Correlated theta- and ripple-coupling also indicate a common source capable of linking MRR GABAergic activity, and *via* inhibition, a large part of ascending modulation from the MRR to these oscillatory patterns. One such source can be the medial septum known to project to brainstem modulatory centers, including the median raphe nucleus. Importantly, short timescale ripple-coupling was embedded in multiple second-long alterations of spiking. The presence and direction of rapid change of activity were not correlated with long timescale modulation. Thus, the two phenomena may be controlled by diverging mechanisms. In summary, our results provide correlative evidence for the role of the GABAergic circuit of the MRR in enabling the rapid segmentation of modulation on a subsecond timescale. This raises the possibility of entirely novel, yet unknown forms of modulation acting on high temporospatial resolution.

## Data Availability Statement

The raw data supporting the conclusions of this article will be made available by the authors, without undue reservation.

## Ethics Statement

The animal study was reviewed and approved by the Animal Care and Use Committee of the Institute of Experimental Medicine and the Committee for Scientific Ethics of Animal Research of the National Food Chain Safety Office of Hungary.

## Author Contributions

MJ, AB, and VV designed the experiments. MJ and FK carried out experiments. MJ and AB performed the analysis. MJ and VV wrote the original manuscript. AB and VV acquired the funding. All authors contributed to the discussion and interpretation of the results.

## Conflict of Interest

The authors declare that the research was conducted in the absence of any commercial or financial relationships that could be construed as a potential conflict of interest.

## Publisher’s Note

All claims expressed in this article are solely those of the authors and do not necessarily represent those of their affiliated organizations, or those of the publisher, the editors and the reviewers. Any product that may be evaluated in this article, or claim that may be made by its manufacturer, is not guaranteed or endorsed by the publisher.

## Footnotes

1
open-ephys.org

2
sites.google.com/site/tarotoolsregister
