# Supplementary material for: Activity and Coupling to Hippocampal Oscillations of Median Raphe GABAergic Cells in Awake Mice
Source: Front Neural Circuits. 2021 Dec 17;15:784034. doi: 10.3389/fncir.2021.784034 (PMC8718440; doi:10.3389/fncir.2021.784034)
Supplement: Supplementary file 1 [file Data_Sheet_1.pdf]

## Supplementary Material

### Supplementary Figures and Tables

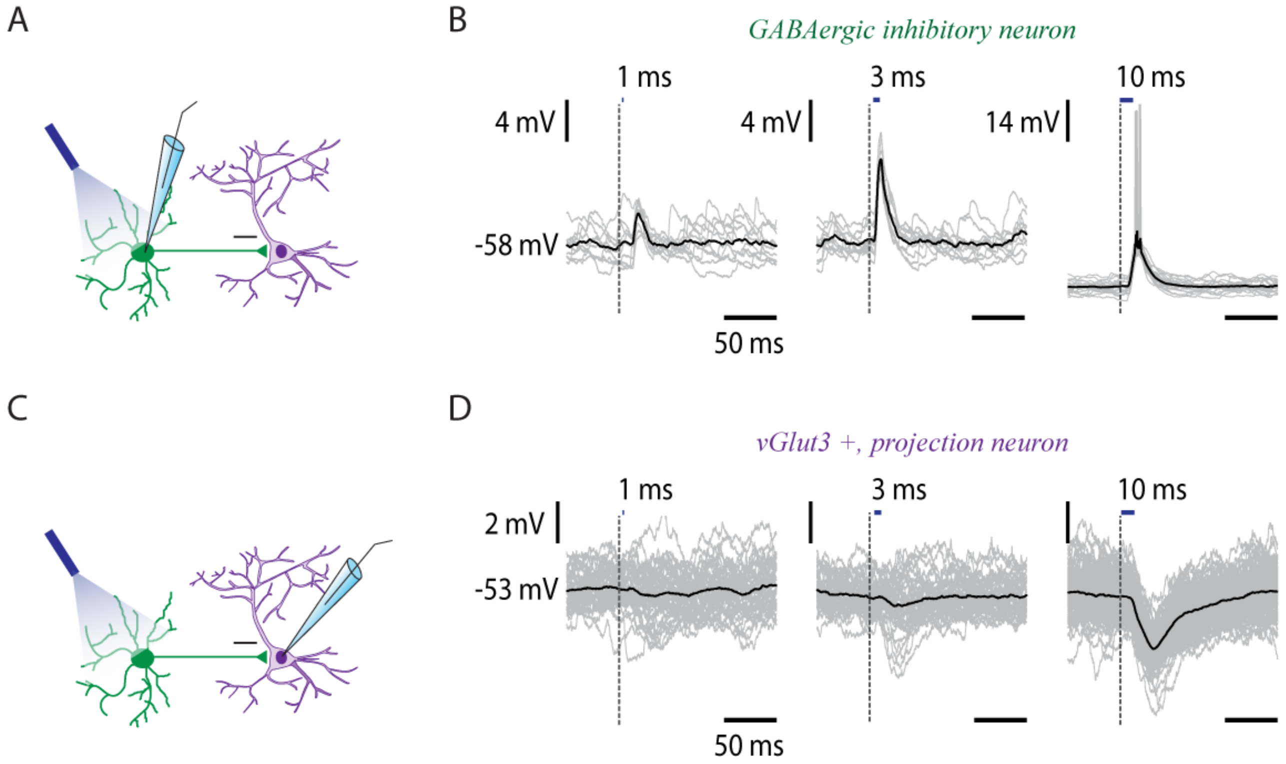


**Supplementary Figure 1 Light activation of MRR GABAergic neurons**

(A, C) Schematic showing recording configuration during optogenetic stimulation of GABAergic cells (ChR2, 447 nm). (B) 1-3-10 ms long stimulus aligned voltage traces from a representative GABAergic cell. Grey traces represent the individual voltage traces and black line represents the Vm average. (D) 1-3-10 ms long stimulus aligned voltage traces from a representative non-GABAergic cell. Grey traces represent the individual voltage traces and black line represents the Vm average.

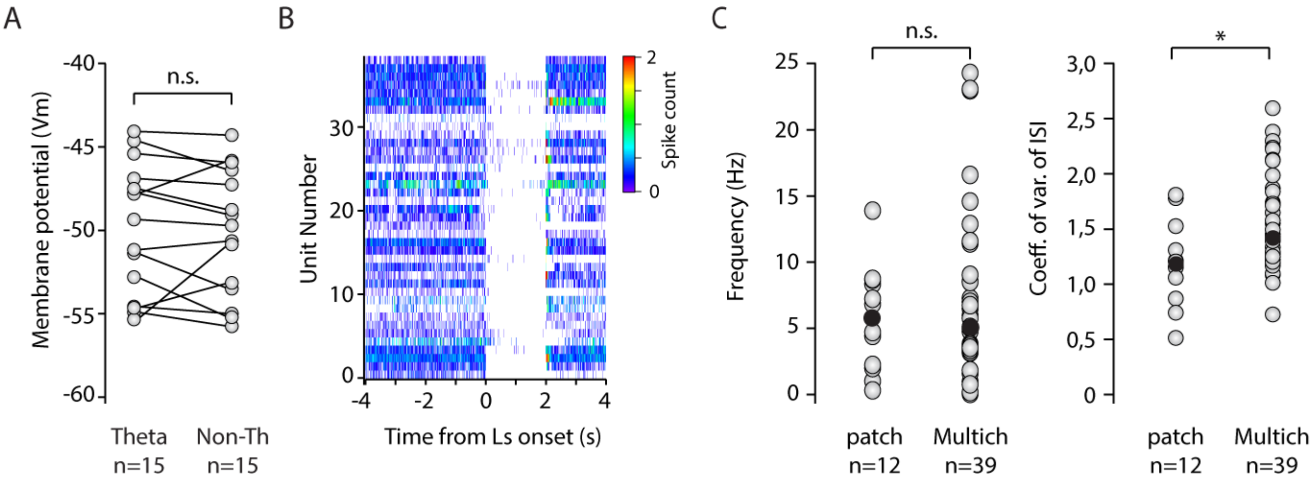


**Supplementary Figure 2**

(A) Average membrane potentials of whole cell recorded MRR neurons (n=15) during spontaneous theta (Theta) and non-theta segments (Non-Th). n.s.= not significant, Wilcoxon signed rank test (B) Laser onset (2s, 593 nm) triggered peristimulus time histograms of 39 MRR neurons, bin size 10ms. (C) Average firing rates (left) and coefficient of variations of inter-spike intervals (right) of whole cell patch-clamp recorded and identified GABAergic cells (patch, n=12) and multichannel recorded GABAergic cells (Multich, n=39). n.s.=not significant, *p<0.05, Wilcoxon signed rank test.

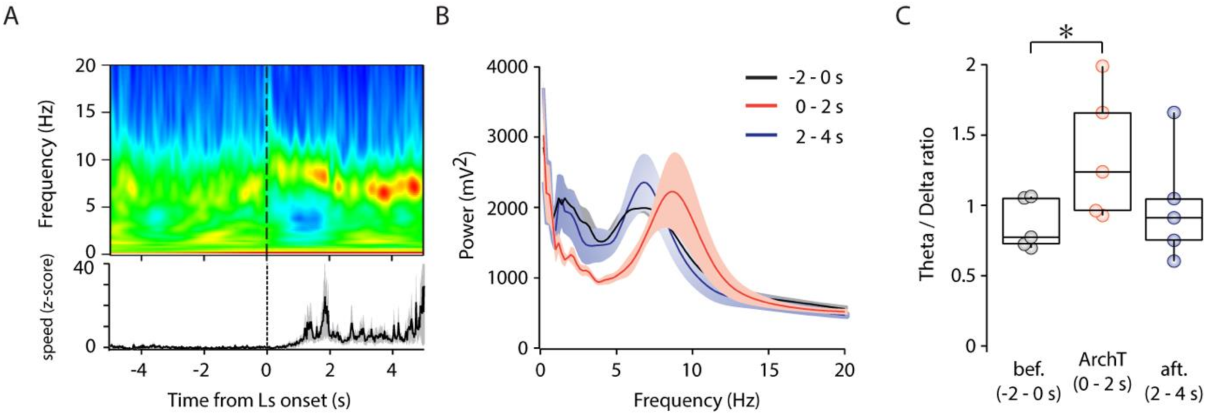


**Supplementary Figure 3 LFP changes coupled to MRR GABAergic cells suppression**

(A) Stimulus-triggered wavelet of theta and sub-theta bands during light-induced (593 nm, ArchT, 2s) suppression of GABAergic cells and the accompanying averaged speed (n=5). (B) Power spectra in the 0 – 20 Hz range before (-2 - 0 s), during (0 - 2 s) and after light stimulations (2 – 4 s, ArchT, 593 nm) silencing presumably the majority of GABAergic cells. (C) Ratio of theta (5-12 Hz) and delta (1-4 Hz) bands power before (-2 - 0 s), during (0 - 2 s) and after light delivery (2 – 4 s), ArchT, 593 nm), *p<0.05, two-tailed paired *t*-test.

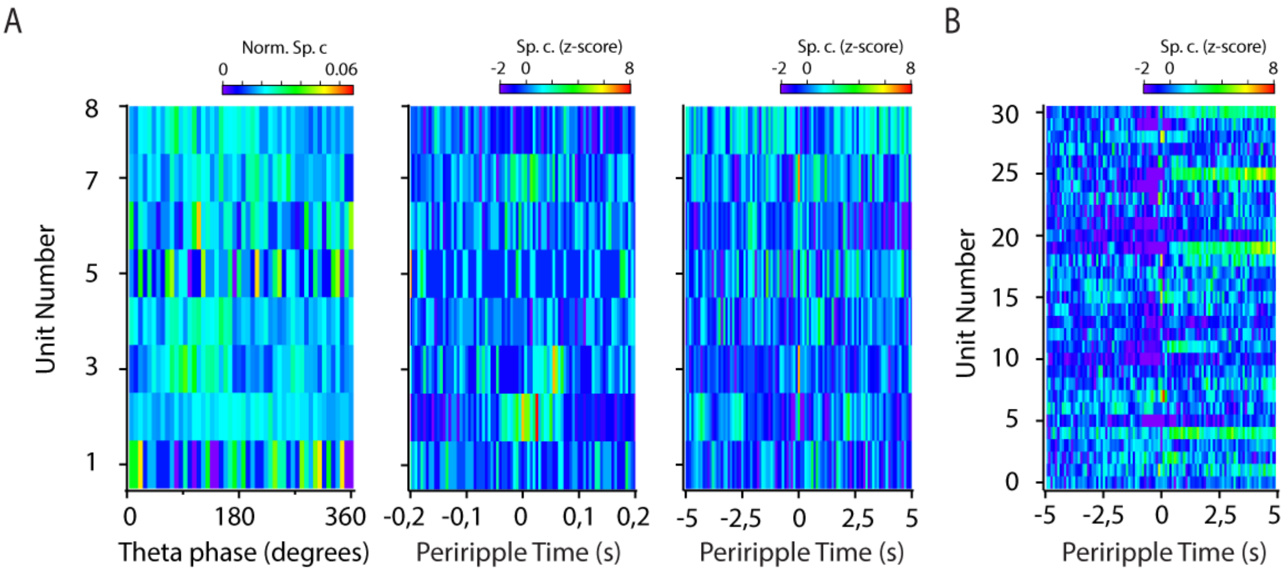


**Supplementary Figure 4 Ripple-coupled firing of GABAergic cells with no theta preference**

(A) Preferred (non-significantly) theta phase-ordered phase histograms (left panel) and corresponding peripripple firing histograms of GABAergic cells with no theta preference (n=8) in 200 ms and 5 second-long windows (B) Peripripple firing histograms of theta coupled GABAergic cells (n=31) on 5 second-long timescale.

**Supplementary Table 1 Electrophysiological properties of whole cell (WC) and cell-attached (CA) patch-clamp recorded MRR cells from awake mice.** Frequency, Inter-spike intervals and CV of ISIs are presented in median and interquartile range (25-75%). Vm, SD of Vm and threshold are presented in mean ± s.d. Duration of recordings is presented in mean ± s.d and range of values.

| | GABAergic cells | Non-GABAergic cells | Non-identified cells |
|---|---|---|---|
| Frequency (Hz) | 5.78 (2.19–8.43) | 5.54 (3.17 – 17.15) | 7.41 (3.23 – 14.15) |
| Inter-spike interval (ms) | 145.95<br>(118.12 – 457.87) | 179.70<br>(58.33 – 315.3) | 134.2<br>(70.67 – 308.95) |
| Coeff. of var. (ISI) | 1.18 (1.01-1.36) | 1.11 (0.67-1.66) | 1.11 (0.79-1.61) |
| Recording (CA+WC) length (s) | 110.5 ± 102<br>(34 - 340) | 191.5 ± 139<br>(66 - 508) | 254 ± 191<br>(30 - 978) |
| Number (CA+WC) | n = 12 | n = 13 | n = 83 |
| Membr. pot (Vm) | -49.5 ± 4.03 | -48.69 ± 4.03 | -49.76 ± 3.93 |
| SD of Vm | 3.56 ± 0.22 | 3.04 ± 0.52 | 3.76 ± 1.74 |
| Threshold (Vm) | -33.77 ± 3.42 | -35.60 ± 6.88 | -34.95 ± 6.92 |
| WC recording length (s) | 113.4 ± 87.5<br>(34 - 250) | 177.5 ± 105<br>(80 - 379) | 153.8 ± 134.9<br>(47 - 500) |
| Number (WC) | n = 7 | n = 6 | n = 14 |
